# Tunable Dielectric Properties of Poly(vinylidenefluoride-co-hexafluoropropylene) Films with Embedded Fluorinated Barium Strontium Titanate Nanoparticles

**DOI:** 10.1038/s41598-018-22442-2

**Published:** 2018-03-06

**Authors:** Wooje Han, Taehee Kim, Byungwook Yoo, Hyung-Ho Park

**Affiliations:** 1 Department of Materials Science and Engineering, Yonsei University, 50 Yonsei-ro, Seodaemun-gu, Seoul, 03722 Republic of Korea; 2 Flexible Display Research Center, Korea Electronics Technology Institute, Seongnam, Gyeonggi 13509 Republic of Korea

## Abstract

Fluoropolymer nanocomposites of poly(vinylidene fluoride-co-hexafluoropropylene) (PVdF-HFP) were prepared using fluorinated barium strontium titanate (Ba_1−x_Sr_x_TiO_3_, BSTO) nanoparticles (NPs) by low-temperature synthesis using a modified liquid–solid solution process. The exact stoichiometry of as-synthesized BSTO NPs was confirmed by X-ray diffraction analysis along with lattice parameter calculations. The synthesized BSTO NPs were fluorinated using 2,2,2-trifluoroacetic acid as a fluorous ligand. The BSTO NPs showed high solubility in the fluorous system (polymer and solvent) on account of their modified surface. The root-mean-square roughness of the fluorinated BSTO/PVdF-HFP nanocomposite was 76 times lower than that of the nonfluorinated BSTO/PVdF-HFP nanocomposite. The dielectric constant of the fluorinated BSTO/PVdF-HFP nanocomposite exhibited Curie temperature behavior. The dielectric constant of the nanocomposite predicted using the modified Kerner model at room temperature agreed well with the experimental values.

## Introduction

Poly(vinylidene fluoride-co-hexafluoropropylene) (PVdF-HFP) is a fluoropolymer with attractive properties. It is less coarse and shows superhydrophobicity with a low surface energy^1^. Its crystalline chain comprising vinylidene fluoride shows remarkable chemical resistance and mechanical strength, whereas its amorphous chain made of hexafluoropropylene has good elasticity and ionic conductivity. Furthermore, a high dielectric constant of PVdF-HFP (k = 8.4) induces a high charge carrier concentration^2^.

High-k barium strontium titanate (Ba_1−x_Sr_x_TiO_3_, BSTO) has been widely investigated owing to its good ferroelectricity^3^ (e.g., high dielectric constant and high nonlinear optical coefficient)^4^, which is strongly dependent on factors such as composition, crystalline size, and crystal structure. Bulk BaTiO_3_ has a high dielectric constant, and it is considered a suitable material for several electronic applications such as multilayer capacitors, condensers, positive-temperature-coefficient thermistors, piezoelectric transducers, and various electro-optic devices. BaTiO_3_ also shows a paraelectric-to-ferroelectric transition with the highest permittivity peak at 130 °C^5^. However, partial substitution of Ba sites with other isovalent cations (e.g., strontium) alters their dielectric properties, and the highest permittivity peak moves toward lower temperatures. Atomic substitution not only affects the condition of the ferroelectric transition but also broadens the phase transition significantly, which promotes its application to various electronic devices^5^.

The need for high-performance dielectric materials in electronic devices has led to investigation of the fluoropolymer nanocomposite, which combines the breakdown field strength and processability of a fluoropolymer with the high dielectric constant of oxide nanoparticles^6^. Attractively, inorganic high-k nanoparticles (NPs) enhance the effective dielectric properties of nanocomposites without degrading the high intrinsic dielectric strength of fluoropolymers^7^. To improve the dielectric properties of nanocomposites, e.g., to increase their dielectric constant and reduce the dielectric loss, it is essential for NPs to have a homogeneous distribution and high crystallinity^7^. High-k materials can be used to store energy. Nanocomplexation studies have been conducted to investigate the high dielectric constant of such NPs^8–10^. Inorganic oxide NPs with high crystallinity and a narrow size distribution can be synthesized by the liquid–solid solution (LSS) process; however, the LSS process requires solvothermal treatment above 180 °C under high pressure for a long period and the use of expensive metal alkoxide precursors^11^. Oxide NPs with a uniform size and high crystallinity can be obtained in air atmosphere at low temperatures by using a modified LSS process^12^. In spite of the tunable dielectric properties of barium strontium titanate, it has some limitations for application as a fluoropolymer composite in which a high-k material can be embedded. For example, it is difficult to prepare fluorous complexes by using oxide NPs synthesized by the organic solution process because of the poor dispersion of these NPs in fluorous systems^13,14^; this can be remedied by achieving fluorous ligand exchange on the surface of inorganic NPs. Fluorous ligand exchange has been performed to study the effects of fluorination between high-k inorganic NPs and fluorous ligands (such as pentafluorobenzyl phosphonic acid and trifluoroacetic acid)^15,16^. Herein, fluoropolymer nanocomposites are synthesized using PVdF-HFP and high-k NPs, and they exhibit Curie temperature behavior, a high dielectric constant, low dielectric loss, and very low surface roughness. Effective permittivity of the synthesized nanocomposites is simulated using the modified Kerner model, which was developed to determine the dielectric constants of nanocomposites^17^.

## Results and Discussion

### Barium strontium titanate NPs

**Figure 1:**
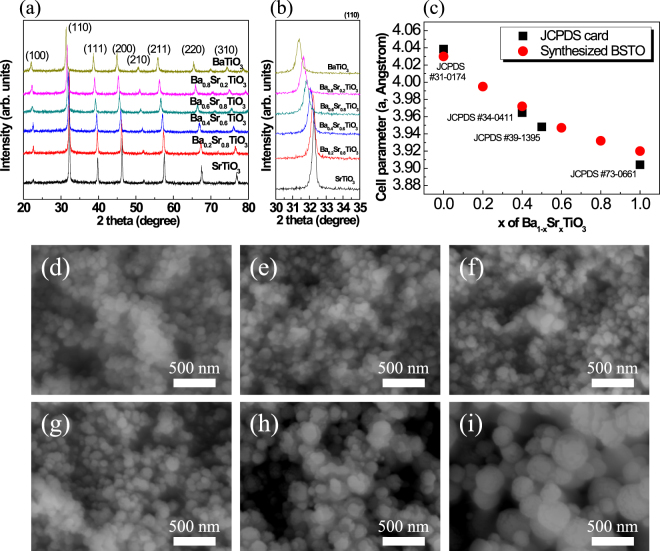
(**a**) XRD pattern, (**b**) (110) plane of XRD pattern, and (**c**) cell parameters of as-synthesized BSTO NPs, in comparison with JCPDS card values. SEM images of BSTO NPs prepared with different stoichiometries: (**d**) BaTiO_3_, (**e**) Ba_0.8_Sr_0.2_TiO_3_, (**f**) Ba_0.6_Sr_0.4_TiO_3_, (**g**) Ba_0.4_Sr_0.6_TiO_3_, (**h**) Ba_0.2_Sr_0.8_TiO_3_, and (**i**) SrTiO_3_.

BSTO NPs were synthesized using a modified LSS process under refluxing conditions in air. Figure 1(a) and (b) show XRD patterns of the BSTO NPs. A comparison with the values in reference JCPDS card nos. 31-0174^18^ and 73-0661^19^ revealed that both BaTiO_3_ NPs and SrTiO_3_ NPs have the cubic phase. The (110) XRD patterns (Fig. 1(b)) show that for BSTO, 2θ increased with increasing Sr content owing to substitution of the smaller Sr at the sites of the larger Ba. The calculated lattice parameters of the BSTO NPs agreed well with the lattice parameters in the JCPDS cards (Fig. 1(c)), confirming that Ba and Sr were homogeneously mixed with excellent stoichiometry. SEM images of the synthesized BSTO NPs for different values of x in Ba_1−x_Sr_x_TiO_3_ are shown in Fig. 1(d–i). The BaTiO_3_ NPs synthesized by the modified LSS process had diameters of 110–130 nm, whereas the synthesized SrTiO_3_ NPs had diameters of 220–410 nm owing to the higher reactivity of the Sr precursor than that of the Ba precursor in solution. BSTO NPs with a higher Ba content showed a gradual decrease in size to less than 200 nm with a narrow size distribution. The particle size variations of BSTO with the values of x in Ba_1−x_Sr_x_TiO_3_ as determined from the XRD and SEM results are given in Table S1. The BSTO NPs were found to have an average size of 110–470 nm. The particle size distribution of the synthesized BSTO NPs is shown in Fig. S8. Analysis using a particle size analyzer (PSA) showed that the particle size distribution of the nanoparticles was narrow. A reduction in particle size with increasing Sr content was observed in both the XRD and the SEM measurements. The higher concentration of sodium hydroxide enhanced the stability of the NPs through a high basicity effect, which enabled the isolation of NPs and their uniform dispersion in an aqueous medium. The formation of BSTO NPs was also confirmed by FT-IR analyses; the results are shown in Figs S1 and S2. The dielectric constant of the as-synthesized BSTO was measured to be 122–135 at 1 MHz by using BSTO NP pellets prepared at room temperature (Table 1). The dielectric constant of nanomaterials decreases with decreasing crystal size during the formation of NPs^20^. Furthermore, the surface energy of the nanomaterials increases, which results in a larger lattice constant^21^. These phenomena lower the dielectric constant of nanomaterials^22^. Figure 1(c) shows that the synthesized BaTiO_3_ NPs have a slightly larger lattice constant than that provided in the JCPDS card. It has been reported that the above-discussed effects lead to a low dielectric constant. According to JCPDS #31-0174, BaTiO_3_ does not have a very high dielectric constant^23^. In contrast, SrTiO_3_ NPs larger than 400 nm have an even smaller lattice constant. Therefore, large, densified SrTiO_3_ NPs could have a higher dielectric constant because the size of SrTiO_3_ NPs is four times that of BaTiO_3_ NPs. Nanocomposites with high dielectric constants can be achieved using these NPs. The dielectric constant of BSTO depends on its composition because the composition-dependent Curie temperature is the point at which the dielectric properties of certain materials change; at this temperature, the dielectric constant shows abrupt changes. The Curie temperature of Ba_0.6_Sr_0.4_TiO_3_, which is the composition having the highest dielectric constant, is close to room temperature^5^.

**Table 1:** Dielectric constant and dielectric loss of as-synthesized BSTO NPs at a frequency of 1 MHz (Curie temperature taken from ref.^7^).

	BaTiO_3_	Ba_0.8_Sr_0.2_TiO_3_	Ba_0.6_Sr_0.4_TiO_3_	Ba_0.4_Sr_0.6_TiO_3_	Ba_0.2_Sr_0.8_TiO_3_	SrTiO_3_
Dielectric constant	122 (±1)	124 (±2)	159 (±2)	125 (±3)	113 (±2)	135 (±7)
Dielectric loss	0.031	0.037	0.033	0.034	0.032	0.072
Curie temperature^7^ (K)	390	340	290	200	110	30

### Fluorination of BSTO NPs

**Figure 2:**
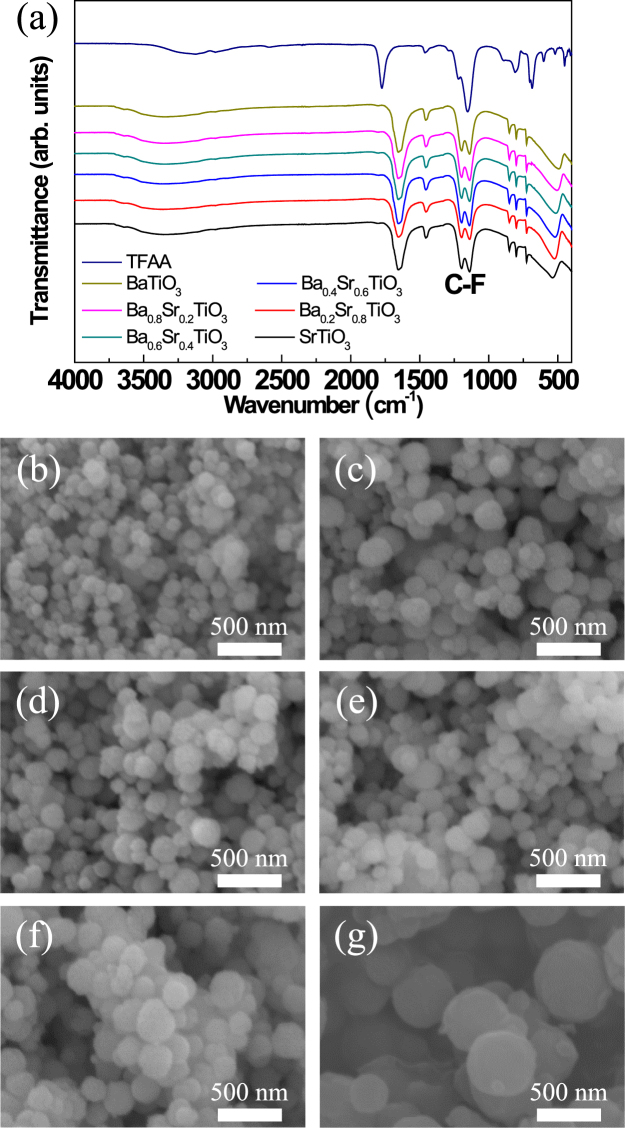
(**a**) FT-IR spectra of fluorinated BSTO NPs prepared using BSTO/TFAA = 2 and SEM images of fluorinated BSTO NPs: (**b**) BaTiO_3_, (**c**) Ba_0.8_Sr_0.2_TiO_3_, (**d**) Ba_0.6_Sr_0.4_TiO_3_, (**e**) Ba_0.4_Sr_0.6_TiO_3_, (**f**) Ba_0.2_Sr_0.8_TiO_3_, and (**g**) SrTiO_3_.

BSTO NPs were fluorinated via ligand exchange using TFAA. Figure 2(a) shows FT-IR spectra of fluorinated BSTO NPs with various stoichiometric compositions at a BSTO NP/TFAA ratio of 2. In a previous study, fluorinated BaTiO_3_ NPs with a BSTO NP/TFAA ratio of 2 were confirmed to have adequate bonded fluorous ligands on the NP surfaces; thus, BSTO NP/TFAA = 2 was selected as the optimum ratio for the fluorination of BSTO NPs^12^. The C-F bond peak at 1300 cm^−1^ corresponds to asymmetric F-C-F stretching at high wavenumbers and to symmetric F-C-F stretching at low wavenumbers, owing to better interaction between the fluorous ligands and the NPs under sufficient thermal energies^16^. As the acid group of TFAA was bound to the NP surface and the symmetric C-F stretching was restricted by steric hindrance, the peak intensity of the symmetric stretching decreased. The C=O stretching band (1750 cm^−1^) in the spectrum of pure TFAA was absent in the spectrum of fluorinated BSTO; however, new bands due to symmetric ν_s_(COO–) stretching appeared at 1650 cm^−1^. This may be because the bonding pattern of the carboxylic acids on the NP surface was a combination of symmetrically bonded molecules and molecules bonded at an angle to the surface^24^. Figure 2(b)–(g) show SEM images of the various fluorinated BSTO NPs synthesized with various Ba-Sr compositions. After fluorination treatment, the size distribution of the NPs became uniform but the size increased from 1 nm to 20 nm owing to the latent heat produced during fluorination. The -OH groups on the surface were replaced with F-containing functional groups, and the size distribution was controlled by F repulsion.

### Fluorinated BSTO/PVdF-HFP nanocomposites

**Figure 3:**
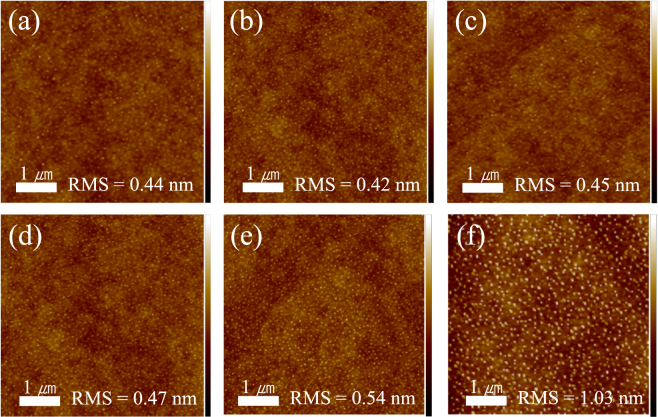
AFM images of fluorinated BSTO/PVdF-HFP nanocomposites: (**a**) BaTiO_3_, (**b**) Ba_0.8_Sr_0.2_TiO_3_, (**c**) Ba_0.6_Sr_0.4_TiO_3_, (**d**) Ba_0.4_Sr_0.6_TiO_3_, (**e**) Ba_0.2_Sr_0.8_TiO_3_, and (**f**) SrTiO_3_ (the scale ranges from 5 nm (white) to −5 nm (black)).

Figure 3 shows AFM images (5 µm × 5 µm) of the fluorinated BSTO/PVdF-HFP nanocomposites with various Ba-Sr compositions. All the fluoropolymer nanocomposites showed smooth morphologies with low root-mean-square (RMS) roughness values of <1.04 nm. A slight change in the roughness of pristine PVdF-HFP upon the addition of the fluorinated NPs was confirmed; this change occurred owing to the bonding between a fluorinated NP and the fluoropolymer matrix., which originated from the interaction between the fluoride group of the fluoropolymer and the fluorous ligand on the fluorinated NPs. The TFAA ligands on the NPs enhanced the stability of the fluoropolymer nanocomposite because of the affinity of the fluorous ligands on the fluorinated BSTO NPs for the fluorine parts in PVdF-HFP. Consequently, the BSTO NPs fluorinated using TFAA were homogeneously dispersed in the fluoropolymer matrix. This also prevented particlization of the polymer and agglomeration of the NPs. The affinity of the fluorine on the NPs for the fluorine chains in the fluoropolymer led to a decrease in the RMS roughness. The RMS roughness of the fluorinated BSTO/PVdF-HFP nanocomposite was controlled to an average value of 0.56 nm, which is slightly higher than that of pristine PVdF-HFP film (0.34 nm) (Fig. S3). The highest RMS roughness of 1.03 nm was obtained for the nanocomposite with STO NPs because of the larger size of these NPs. The flat surface of the fluorinated BSTO/PVdF-HFP nanocomposite was confirmed by SEM images (Fig. S6). The morphology of the fluorinated BSTO/PVdF-HFP nanocomposite was comparable to that observed in the AFM images (Fig. 3). It could be confirmed from the cross-sectional SEM images that the fluorinated nanoparticles were well dispersed in the polymer matrix. Figure S6(l) shows a cross-sectional SEM image of the fluorinated SrTiO_3_/PVdF-HFP nanocomposite. The composite film with the largest BSTO NPs was found to be the thickest. Even the nanocomposite with the largest particles was well dispersed in the polymer matrix owing to fluorination, which was demonstrated via SEM. In contrast, the nontreated NPs were not dispersed in the polymer matrix (Fig. S7). The results indicate that dispersibility could be controlled by fluorinating NPs.

**Figure 4:**
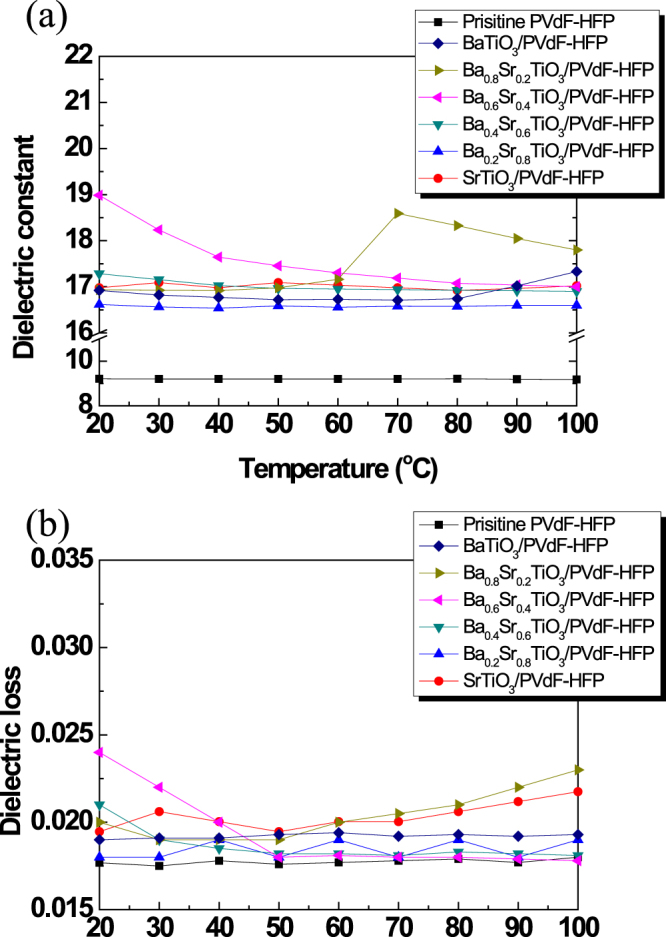
Electrical properties of fluorinated BSTO/PVdF-HFP nanocomposites at various temperatures: (**a**) dielectric constant and (**b**) loss tangent.

The effective dielectric constant of the fluorinated BSTO/PVdF-HFP nanocomposites was determined by C–V measurements of fluoropolymer nanocomposite films with different Ba-Sr compositions. Figure 4(a) shows the dielectric constants of fluorinated BSTO/PVdF-HFP nanocomposites with various Ba-Sr compositions over a wide temperature range at 1 MHz. The real part of the dielectric constant of the fluorinated BSTO/PVdF-HFP nanocomposites was dependent on temperature. The peak of the dielectric constant gradually shifted toward lower temperatures with increasing Sr content (Fig. S4); Ba_0.6_Sr_0.4_TiO_3_ exhibited the highest dielectric constant at 20 °C. Notably, the paraelectric-to-ferroelectric transition is mainly dependent on two factors: (i) chemical composition and (ii) temperature. At the Curie temperature, high-k materials showed the highest value of the dielectric constant. Both an increased dielectric constant and reduced dielectric loss were observed, implying incorporation of the fluorinated BSTO NPs into the nanocomposite.

The dielectric constant of the nanocomposite containing 20 vol.% BSTO NPs increased to 17–19. The dielectric loss is quantified as the dissipation factor. It is defined as the ratio of the imaginary part of the dielectric constant to its real part^25^. The dielectric loss of the nanocomposites as a function of the Ba-Sr composition of the BSTO NPs over a wide temperature range is depicted in Fig. 4(b). Values of the dielectric loss of the fluorinated BSTO/PVdF-HFP nanocomposites were found to be temperature dependent. The imaginary part of the electric modulus indicated a certain loss tangent, which helped in interpreting the relaxation phenomenon. The electric modulus formalism has been used to investigate interfacial polarization in several composites^26^. Since the relaxation time of interfacial polarization is considerably long, the peak of dielectric loss of Ba_0.6_Sr_0.4_TiO_3_ represents interfacial polarization at room temperature. A lower dielectric loss could be an indicator of an enhancement of interfacial polarization. In the absence of the BSTO NPs, the relaxation peak showed the lowest value, indicating that the incorporation of BSTO NPs increased the interfacial polarization in the two types of nanocomposites studied. Apparently, the shift in the relaxation peak was more significant for the fluorinated BSTO/PVdF-HFP nanocomposites. Interfacial polarization in fluorinated Ba_0.6_Sr_0.4_TiO_3_/PVdF-HFP nanocomposites was stronger than that in any other fluorinated BSTO/PVdF-HFP nanocomposite. Similar results have been reported for numerous composite systems^26,27^. For the fluoropolymer nanocomposite with 20 vol.% fluorinated BSTO NPs, a dielectric loss value of ~0.025 was observed at 1 MHz; nevertheless, the loss caused by the incorporation of fluorinated BSTO NPs had a very low value. Malyshikina *et al*. revealed that HFP chains of PVdF-HFP exhibited relaxor ferroelectric behavior^28^ owing to interactions among the NPs. This could explain the semicrystalline structure of the PVdF-HFP fluoropolymer host or disruption of the segmental motion^29^. The experimental results of the dielectric loss showed that it was dependent on the resonance of the matrix material. The dielectric constants calculated using the modified Kerner method (Table 2) were compared with the experimentally determined values for the fluoropolymer nanocomposites.

**Table 2:** Theoretical predictions and experimental values of dielectric constants of fluorinated BSTO/PVdF-HFP nanocomposites at a frequency of 1 MHz.

	Modified Kerner model	Experimental value
BaTiO_3_/PVdF-HFP	16.9	16.7 (±1.1)
Ba_0.8_Sr_0.2_TiO_3_/PVdF-HFP	16.9	16.5 (±1.4)
Ba_0.6_Sr_0.4_TiO_3_/PVdF-HFP	17.5	18.4 (±1.7)
Ba_0.4_Sr_0.6_TiO_3_/PVdF-HFP	17.0	17.2 (±1.9)
Ba_0.2_Sr_0.8_TiO_3_/PVdF-HFP	16.7	16.6 (±1.8)
SrTiO_3_/PVdF-HFP	17.2	16.9 (±2.4)

The experimentally determined dielectric constants of the fluoropolymer nanocomposites increased with increasing dielectric constant of the BSTO NPs. From Table 2, it can be seen that the dielectric constants calculated using the modified Kerner model agreed well with those measured experimentally (see Supporting Information for details of the calculation method).

Furthermore, the surface morphologies and dielectric properties of the fluoropolymer nanocomposites, which were obtained by spin coating of the as-synthesized Ba_0.6_Sr_0.4_TiO_3_ NPs (without fluorination), were studied to further observe the effects of NP fluorination and sonication. The dielectric constants of the nonfluorinated Ba_0.6_Sr_0.4_TiO_3_/PVdF-HFP nanocomposites were low, whereas the dielectric losses were markedly high (Table 3). Despite the application of sonication for fabrication of a thin film, inferior dispersion was observed, which is similar to the results obtained without fluorination. This was due to extensive agglomeration of NPs and granulation of the polymer, as observed by the AFM studies (Fig. S5). Higher RMS roughness values of 35.9 nm and 34.4 nm (in comparison to a value of around 0.45 nm for the fluoropolymer nanocomposites with fluorinated NPs) were confirmed for the nonfluorinated Ba_0.6_Sr_0.4_TiO_3_/PVdF-HFP nanocomposites with 20 vol.% NPs. NP agglomeration was promoted by the presence of -OH ligands on the NPs. The hydroxyl ligands were repelled by the fluorine atoms in PVdF-HFP. These repulsive forces caused close aggregation of NPs. Repulsion and agglomeration of the NPs could have been caused by the phase separation of the fluoropolymer^30^. Therefore, the RMS roughness decreased 76-fold by the fluorination of NPs, irrespective of the application of sonication. This improvement in roughness clearly demonstrates that the homogeneous distribution of NPs in the fluoropolymer matrix is critical for inhibiting granulation and agglomeration. Fluorination of NPs affects not only their dispersion but also their dielectric constant. In order to obtain a high dielectric constant of a nanocomposite, both NPs and polymers must store electric charge between electrodes. However, the commonly as-synthesized BSTO/PVdF-HFP nanocomposite does not store electric charge, because of the agglomeration of particles. These results were observed during the determination of the dielectric constant of the Ba_0.6_Sr_0.4_TiO_3_/PVdF-HFP nanocomposite (Table 3). High dielectric loss was also observed, confirming that the current flowed instead of being stored as electric charge. However, the fluorinated NPs were well distributed in the fluoropolymer matrix, and the electrostatic charge did not leak. The theoretical dielectric constant of the fluoropolymer nanocomposites can be considered depending on the distribution state of NPs in the matrix. When the NP distribution in the matrix is poor, the dielectric properties are worse than the inherent properties of the original polymer despite the addition of NPs. Nanocomposites with good dielectric properties can be used for energy storage and to produce metal oxide-polymer nanocomposites because they have a high energy density^29,31–34^.

**Table 3:** Dielectric constant and dielectric loss of Ba_0.6_Sr_0.4_TiO_3_/PVdF-HFP nanocomposite synthesized by fluorination and sonication at a frequency of 1 MHz.

Nanocomposite	Fluorination	Sonication	Dielectric constant	Dielectric loss	AFM image
Ba_0.6_Sr_0.4_TiO_3_/PVdF-HFP	X	X	2 (±1.9)	11 (±8)	Fig. S5(a)
	X	O	2 (±1.5)	10 (±5)	Fig. S5(b)
Fluorinated Ba_0.6_Sr_0.4_TiO_3_/PVdF-HFP	O	X	18.5 (±1.8)	0.024 (±0.08)	
	O	O	18.4 (±1.7)	0.023 (±0.05)	Fig. 3(c)

**Figure 5:**
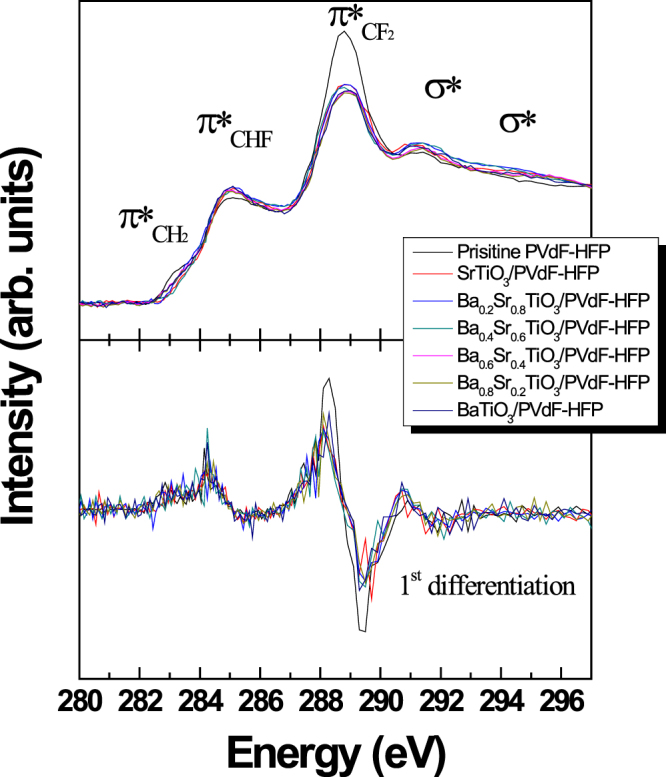
C1s NEXAFS spectra of fluorinated BSTO/PVdF-HFP nanocomposites with different Ba-Sr compositions.

C 1 s NEXAFS analyses were conducted at the 4D and 8A2 beamlines of the Pohang Accelerator Laboratory for understanding the chemical bonding states of the fluoropolymer nanocomposites. C1s NEXAFS spectroscopy is a powerful technique to identify and distinguish small molecules and macromolecules, such as polymers^35^. This technique is extremely sensitive to chemical functional groups; therefore, the obtained pattern can be used as a unique spectroscopic fingerprint to identify specific chemical moieties and functional groups. The photoabsorption cross-section for the excitation of tightly bound core electrons into unoccupied molecular orbitals or the vacuum continuum is measured as a function of photon energy. The detailed spectral features observed in NEXAFS spectra correspond to transitions from the ground state to a core-excited state, where the initial and final states can be manifolds of states for a given element. The complexity of these manifolds depends on the variation in the chemical and hence the electronic environments that can be found in a particular element^36–38^. Because of the complex covalent bonding of carbon atoms in organic materials, carbon 1s NEXAFS is a particularly rich and detailed core absorption edge. Results of the C 1s NEXAFS analyses are shown in Fig. 5. All spectra showed dominant strong C1s → π*_CHF_ and C1s → π*_CF2_ features near 285 eV and 289 eV, respectively^39^. Notably, the fluoropolymer nanocomposites with incorporated fluorinated BSTO NPs showed weak π*_CH2_ signals at 283 eV owing to the diminished CH_2_ bonding state in the polymer, which acted as a carrier element. This chemical bonding state is indirect evidence for interruption of the conduction path via the C1s → π*_CH2_ band^40^. The fluorinated BSTO NPs were finely dispersed in the fluoropolymer matrix. The degree of fluorination of NPs has a significant effect on the dispersion of NPs in the fluoropolymer matrix, and also on the concentration of NPs in the fluoropolymer nanocomposite. The formation of a fine dispersion between the fluorinated NPs and the fluoropolymer resulted in a lowering of the CH_2_ bond state. This fine distribution of the CH_2_ bond is highly useful for investigating its interaction with each dipole owing to densification of the ionic aggregates; these interactions consequently modify the energy states of fluoropolymer nanocomposites^41^. Such a structure was in good agreement with the AFM observations (Fig. 3). It should have an extremely dielectric loss, as shown in Fig. 4. The other parameter, the density of state of π_CF2_, also decreased upon incorporation of the fluorinated BSTO NPs. The surfaces of the fluorinated NPs interacted with the CF_2_ chains in PVdF-HFP, and this resulted in the formation of CHF bonds between the fluorinated BSTO NPs and PVdF-HFP. An increase in the π_CHF_ peak indicates a homogeneous distribution of the inorganic BSTO NPs in the fluoropolymer and consequently a strong synergy between the oxide NPs and PVdF-HFP via CHF bonding.

## Methods

### Synthesis of barium strontium titanate NPs

To synthesize BSTO NPs, barium nitrate (Ba(NO_3_)_2_, 99.999% trace metals basis, Sigma-Aldrich, USA), strontium nitrate (Sr(NO_3_)_2_, 99.995% trace metals basis, Sigma-Aldrich, USA), and titanium (IV) butoxide (Ti(OCH_2_CH_2_CH_2_CH_3_)_4_, reagent grade, 97%, Sigma-Aldrich, USA) were used as the starting materials; n-butanol (99.5%, Duksan, South Korea) and deionized water were used as the solvents; and sodium hydroxide (NaOH, reagent grade, ≥98%, pellets (anhydrous), Sigma-Aldrich, USA) was used as the reactive agent. A modified LSS process with control of the Ba-Sr composition was used (see Supporting Information). The crystalline phase of the BSTO NPs were determined by X-ray diffraction (XRD, Ultima, Rigaku, Japan) analysis using Cu-Kα radiation with a wavelength of 1.5418 Å at 2θ = 20°–80°. A particle size analyzer (PSA, Nanotrac Wave, Microtrac, USA) and a scanning electron microscopy (SEM) apparatus (AIS-2000C, SERON, South Korea) were used to observe the morphology and size distribution of the NPs. Fourier-transform infrared spectroscopy (FT-IR, Perkin Elmer, USA) was employed to confirm the ligands of the BSTO NPs. A current–voltage (C–V) meter (B1500A, Agilent, USA) was used to determine the dielectric properties of the NPs, such as the dielectric constant and loss tangent.

### Modification of fluorous ligands

For the fluorination of the BSTO NPs, 2,2,2-trifluoroacetic acid (TFAA, CF_3_COOH, reagent plus grade, 99%, Sigma-Aldrich, USA) and Novec-7500 (3 M) were used as the fluorinating ligand and fluorination solvent, respectively. Free -OH groups were present on the surfaces of the as-synthesized BSTO NPs. These surface ligands could be treated with fluorous ligands via refluxing of the NPs and TFAA. A fluorination reaction was performed with BSTO/TFAA = 2 at 50 °C^13^. All reactions were performed for 5 h via refluxing in a fluorination solvent. SEM and FT-IR observations were used to confirm the characteristics of the BSTO NPs and to determine the conditions that yielded the fluorination characteristics.

### Processing and characterization of fluorinated BSTO/PVdF-HFP nanocomposites

Poly(vinylidene fluoride-co-hexafluoropropylene) (PVdF-HFP, (CH_2_CF_2_)_x_ [CF_2_CF(CF_3_)]_y_, average M_n_ ≈ 130,000, Sigma-Aldrich, USA) was used as received without further purification as the fluoropolymer matrix. Fluorinated BSTO NPs were dispersed in acetone (10 mL) after sonication for 10 min. Then, 0.5 g of PVdF-HFP was added to the NP solution, and the solution was vigorously stirred for 12 h. The fluoropolymer nanocomposites were spin-coated on aluminum-doped ZnO bottom electrodes. The fluoropolymer nanocomposite films were then rested after being annealed at 120 °C. The filling ratio of the fluorinated BSTO NPs was fixed at 20 vol.%. Aluminum was evaporated on the fluorinated BSTO/PVdF-HFP nanocomposite films as top electrodes by means of a thermal evaporator to determine the dielectric properties dielectric properties, and the C–V measurements were performed using Agilent B1500A. The effective permittivity was simulated using the modified Kerner model, which was developed to determine the dielectric constants of nanocomposites^14^ (see Supporting Information). Surface morphologies of the nanocomposites were examined by atomic force microscopy (AFM, MultiMode 8, Bruker, USA). The chemical bonding state was observed by near-edge X-ray absorption fine structure (NEXAFS) analyses at the 4D and 8A2 beamlines of the Pohang Accelerator Laboratory.

## Electronic supplementary material

Supplemental Information
