# Supplementary material for: Tunable Dielectric Properties of Poly(vinylidenefluoride-co-hexafluoropropylene) Films with Embedded Fluorinated Barium Strontium Titanate Nanoparticles
Source: Sci Rep. 2018 Mar 6;8:4086. doi: 10.1038/s41598-018-22442-2 (PMC5840133; doi:10.1038/s41598-018-22442-2)
Supplement: Supplementary file 1 — Supplemental Information [file 41598_2018_22442_MOESM1_ESM.docx]

Correspondence and requests for materials should be addressed to H.H.P. ([hhpark@yonsei.ac.kr](mailto:hhpark@yonsei.ac.kr))

Supplemental Information for

**Tunable Dielectric Properties of Poly(vinylidenefluoride-co-hexafluoropropylene) Films with Embedded Fluorinated Barium Strontium Titanate Nanoparticles**

Wooje Han^1^, Taehee Kim^1^, Byungwook Yoo^2^, and Hyung-Ho Park^1^*

^1^Department of Materials Science and Engineering, Yonsei University, 50 Yonsei-ro, Seodaemun-gu, Seoul 03722, Republic of Korea
^2^Flexible Display Research Center, Korea Electronics Technology Institute, Seongnam, Gyeonggi 13509, Republic of Korea

**Synthesis of barium strontium titanate NPs**

The (Ba+Sr):Ti molar ratio was kept constant at 1:1 at a concentration of 0.2 mol/L. Sodium hydroxide and titanium butoxide were dissolved in water and n-butanol, respectively. These two solutions were combined, after which barium nitrate and strontium nitrate were dissolved in water and the obtained solution was added to the sodium hydroxide/titanium butoxide solution. The resultant solution was then refluxed at 80 °C for 2 h in air. The precipitate formed in the reaction mixture was separated using a centrifuge at 20,000 rpm for 10 min, and the recovered particles were washed several times with distilled water and n-butanol to remove any remaining organic materials and impurities. Finally, the Ba_x_Sr_(1-x)_TiO_3_ particles were dried at 50 °C for 12 h in an open-air oven.

**Calculation of effective permittivities of nanocomposites**

The modified Kerner model of the dielectric complex was developed to determine the dielectric permittivity of a nanocomposite. Dielectric permittivity can be expressed as $\varepsilon_{\mathrm{eff}}=\varepsilon^{'}+i\varepsilon”$, where the real ($\varepsilon^{'}$) and imaginary ($\varepsilon”$) parts are positive components of the dielectric constant and dielectric loss, respectively. Simple composite theories such as the Kerner model [S1] treat the dielectric filler particles as polarizable spheres, whose dipoles are equivalent to ε_eff_ and are induced under an applied electric field. Electric field reduction within the high-permittivity filler particles is considered; however, the effect of the particles on the field in the host medium is neglected. The effective permittivity is calculated using an average of the permittivities of the matrix and filler particles. The contributions are weighted by the volume fraction and the field for each component, as follows:

$$\varepsilon_{eff}=\frac{\varepsilon_{m}v_{m}+\varepsilon_{f}v_{f}(\frac{E_{fz}}{E_{mz}})}{v_{m}+v_{f}(\frac{E_{fz}}{E_{mz}})}$$

(1)

where $\varepsilon_{m}$ and $\varepsilon_{f}$ are the permittivites and $v_{m}$ and $v_{f}$ are the volume fractions of the matrix and the filler, respectively, and $E_{mz}{\mathrm{and}E}_{fz}$ are the fields for the respective components.

Jayasundere and Smith developed a modified Kerner model [S2] for determining the dielectric constant of a binary composite by considering the interactions between adjacent spheres. The equation of this model is as follows:

$$\varepsilon_{eff}=\frac{\varepsilon_{m}v_{m}+\varepsilon_{f}v_{f}\left[ \frac{3\varepsilon_{m}}{\varepsilon_{f}+2\varepsilon_{m}} \right]\left[ 1+\frac{3v_{f}\left( \varepsilon_{f}-\varepsilon_{m} \right)}{\varepsilon_{f}+2\varepsilon_{m}} \right]}{v_{m}+v_{f}\left[ \frac{3\varepsilon_{m}}{\varepsilon_{f}+2\varepsilon_{m}} \right]\left[ 1+\frac{3v_{f}\left( \varepsilon_{f}-\varepsilon_{m} \right)}{\varepsilon_{f}+2\varepsilon_{m}} \right]}$$

(2)

The effective permittivities of the fluorinated BSTO/PVdF-HFP nanocomposites were determined by measuring the dielectric constants of nanocomposite films with different Ba-Sr compositions. The dielectric constants (ε_r_) were calculated using the relationship

$$\varepsilon_{r}=\frac{c\times d}{\varepsilon_{r}\times A}$$

(3)

where c is the capacitance; d, the thickness; ε_0_, the permittivity in air; and A, the electrode area.

Table S1 presents the sizes of the Ba_1-x_Sr_x_TiO_3_ NPs. The particle size variation of BSTO with x in Ba_1-x_Sr_x_TiO_3_ was obtained from the XRD and SEM results.

Table S1. Sizes of Ba_1-x_Sr_x_TiO_3_ NPs

| (nm) | BaTiO_3_ | Ba_0.8_Sr_0.2_TiO_3_ | Ba_0.6_Sr_0.4_TiO_3_ | Ba_0.4_Sr_0.6_TiO_3_ | Ba_0.2_Sr_0.8_TiO_3_ | SrTiO_3_ |
| --- | --- | --- | --- | --- | --- | --- |
| XRD (FWHM) | 32 | 30 | 29 | 34 | 40 | 52 |
| SEM | 110 | 138 | 140 | 157 | 247 | 472 |

Figure S1 shows FT-IR spectra of the as-synthesized BSTO NPs. The absorption peak of the -OH group was observed at 3,400 cm^-1^ [S3]. The formation of BSTO was confirmed by the presence of a Ti-O octahedral bond absorption peak at around 500 cm^-1^ [S4]. All FT-IR spectra of BSTO show similar trends owing to the formation of a Ti-O octahedron with a gradual shift of the position of the Ti-O absorption peak with a change in the x values in Ba_1-x_Sr_x_TiO_3_ (Fig. S2) and the formation of -OH groups on the surface by the modified LSS method. The Ti-O region (500 cm^-1^) is affected by bonding of O with Ba and Sr.. The Ti-O bond length in Ti-O-Ba_1-x_Sr_x_ depends on x because of the different atomic sizes of Ba and Sr. An increase in Ba content leads to an increase in the size of the unit lattice and increased Ti-O bond distance, which consequently results in a shift of the peak position to lower wavenumbers [S5]. This gradual shift of the peak position indicates homogeneous phase formation of BSTO, as ****confirmed by the XRD data.

Figure S1. FT-IR spectra of as-synthesized BSTO NPs.

Figure S2. FT-IR spectra of as-synthesized BSTO NPs (around 500 cm^-1^).

Figure S3 shows the AFM data of pristine PVdF-HFP films. The RMS roughness of the as-coated polymer was 0.34 nm.

Figure S3. AFM image of pristine PVdF-HFP polymer (the scale ranges from 5 nm (white) to -5 nm (black)).

Figure S4 shows the dielectric constants of the various Ba_1-x_Sr_x_TiO_3_ NPs with different Ba-Sr compositions over a wide temperature range at 1 MHz. The maximum permittivity gradually shifted toward lower temperatures with increasing Sr content.

Figure S4. Dielectric properties of fluorinated BSTO NPs at various temperatures: (a) dielectric constant and (b) loss tangent.

Figure S5 shows extensive agglomeration of NPs and granulation of the polymer. Much higher RMS roughness values of 35.9 nm and 34.4 nm (compared to around 0.45 nm for the nanocomposites with treated NPs) were obtained for the untreated Ba_0.6_Sr_0.4_TiO_3_/PVdF-HFP nanocomposites with 20 vol.% NPs.

Figure S5. AFM images of PVdF-HFP nanocomposites prepared using as-synthesized Ba_0.6_Sr_0.4_TiO_3_ NPs: (a) without fluorination and without sonication and (b) without fluorination and with sonication (the scale ranges from 150 nm (white) to -150 nm (black)).


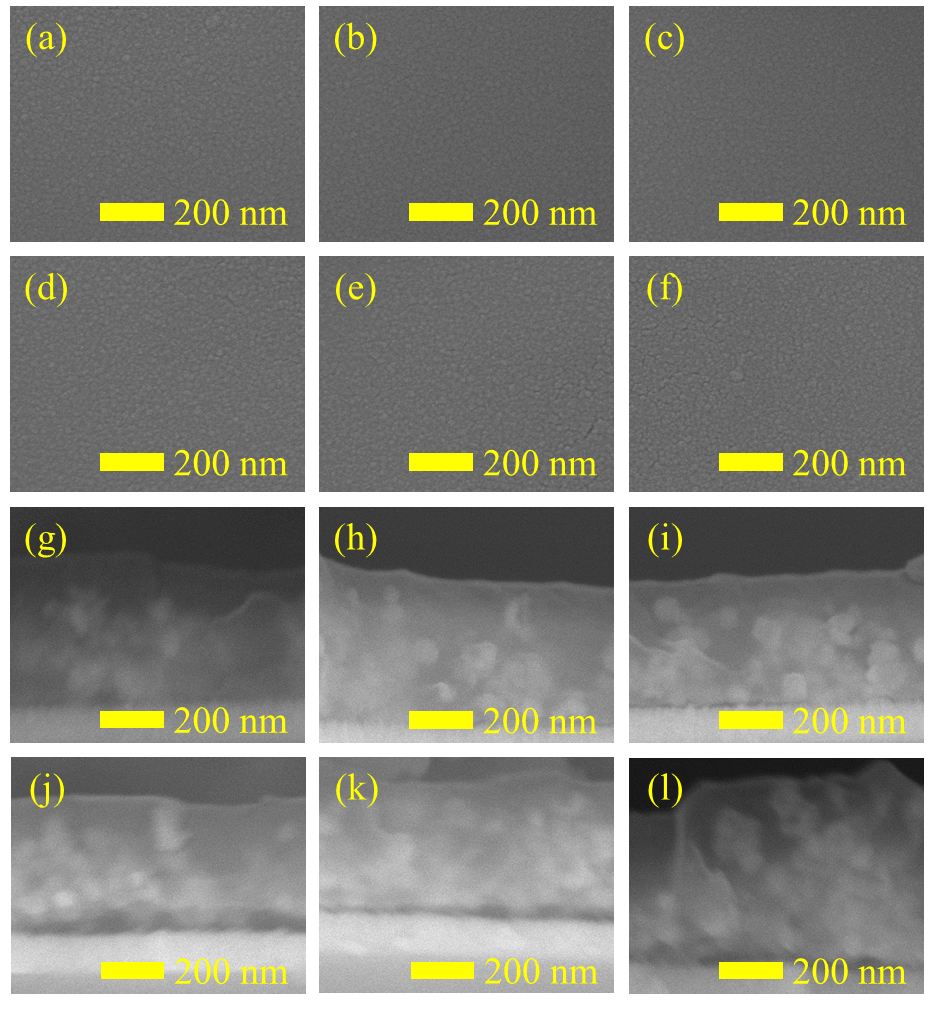
Figure S6. SEM images of fluorinated BSTO/PVdF-HFP nanocomposites: (a) BaTiO_3_, (b) Ba_0.8_Sr_0.2_TiO_3_, (c) Ba_0.6_Sr_0.4_TiO_3_, (d) Ba_0.4_Sr_0.6_TiO_3_, (e) Ba_0.2_Sr_0.8_TiO_3_, and (f) SrTiO_3_. Cross-sectional SEM images of fluorinated BSTO/PVdF-HFP nanocomposites: (g) BaTiO_3_, (h) Ba_0.8_Sr_0.2_TiO_3_, (i) Ba_0.6_Sr_0.4_TiO_3_, (j) Ba_0.4_Sr_0.6_TiO_3_, (k) Ba_0.2_Sr_0.8_TiO_3_, and (l) SrTiO_3_.


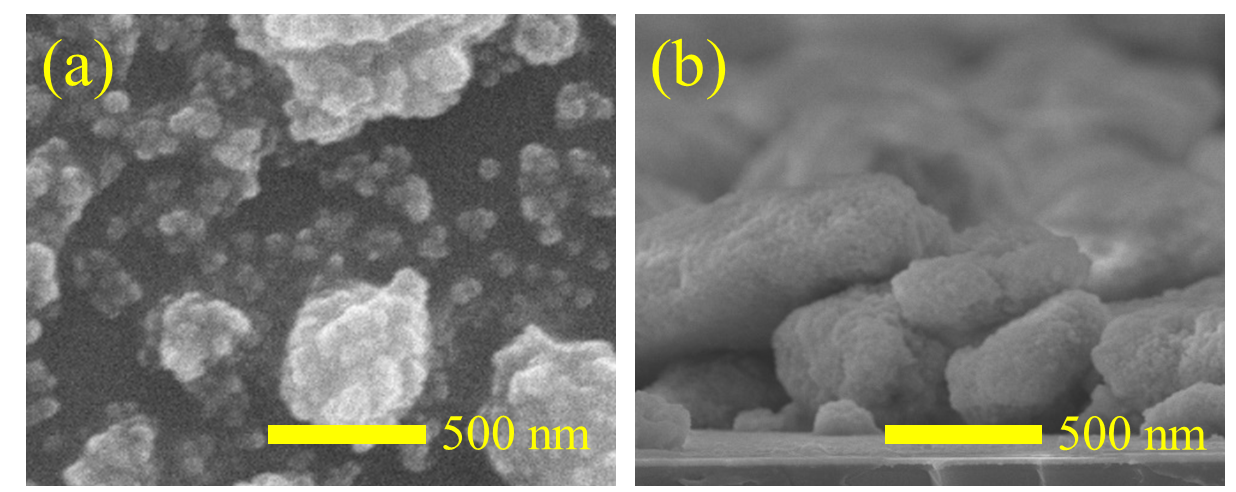
Figure S7. (a) Surface and (b) cross-sectional SEM images of as-synthesized Ba_0.6_Sr_0.4_TiO_3_/PVdF-HFP nanocomposite.

Figure S7 shows SEM images of the as-synthesized Ba_0.6_Sr_0.4_TiO_3_/PVdF-HFP nanocomposite. A rough surface of the nanocomposite was confirmed, and the cross-sectional SEM image showed heterogeneous nanocomplexation of PVdF-HFP with the BSTO NPs. The SEM images were found to be similar to the AFM images (Fig. S5). SEM images of the other BSTO/PVdF-HFP nanocomposites could not be obtained, because of the sample charging problem and considerable differences in the measurement conditions. However, it was possible to compare the surface morphologies of the as-synthesized BSTO/PVdF-HFP nanocomposites and the Ba_0.6_Sr_0.4_TiO_3_/PVdF-HFP nanocomposite during operation of the SEM apparatus. Moreover, the other as-synthesized BSTO/PVdF-HFP nanocomposites showed poorer morphologies than the Ba_0.6_Sr_0.4_TiO_3_/PVdF-HFP nanocomposite.

Figure S8. Particle size distribution of BSTO nanoparticles: (a) BaTiO_3_, (b) Ba_0.8_Sr_0.2_TiO_3_, (c) Ba_0.6_Sr_0.4_TiO_3_, (d) Ba_0.4_Sr_0.6_TiO_3_, (e) Ba_0.2_Sr_0.8_TiO_3_, and (f) SrTiO_3_.

**REFERENCES**

1. A. E. Souza, R. A. Silva, G. T. A. Santos, S. R. Teixeira, S. G. Antonio, M. L. Moreira, D. P. Volanti and E. Longo, Order–disorder degree of self-assembled clusters: Influence on photoluminescence emission and morphology of Ba_x_Sr_1−x_TiO_3_ nanocrystals, Chem. Phys. Lett., 514 (2011) 301-306.
2. E. H. Kerner, The Electrical Conductivity of Composite Media, Proc. Phys. Soc. London Sec. B 69 (1956) 802.
3. N. Jayasundere, B. V. Smith, Dielectric constant for binary piezoelectric 0‐3 composites, J. Appl. Phys. 99 (1993) 2462.
4. S. J. Chang, W. S. Liao, C. J. Ciou, J. T. Lee, C. C. Li, An efficient approach to derive hydroxyl groups on the surface of barium titanate nanoparticles to improve its chemical modification ability, J. Colloid. Interface Sci. 329 (2009) 300-305
5. S. Hao, D. Fu, J. Li, W. Wang, B. Shen, Preparation and characterization of Ag-doped BaTiO_3_ conductive powders, Int. J. Inorganic chemistry 2011 (2011) 837091.
